# The skills related to the early reading acquisition in Spain and Peru

**DOI:** 10.1371/journal.pone.0193450

**Published:** 2018-03-05

**Authors:** Pilar Sellés, Vicenta Ávila, Tomás Martínez, Liz Ysla

**Affiliations:** 1 Department of Occupation Science, Speech Therapy and Developmental and Educational Psychology, Universidad Católica de Valencia, Valencia, Spain; 2 Department of Developmental and Educational Psychology, Universitat de Valencia, Valencia, Spain; 3 Private Teachers College with Official certification "Quality in Educational Networks”. Lima, Perú; Waseda University, JAPAN

## Abstract

This paper deals with the skills related to the early reading acquisition in two countries that share language. Traditionally on reading readiness research there is a great interest to find out what factors affect early reading ability, but differ from other academic skills that affect general school learnings. Furthermore, it is also known how the influence of pre-reading variables in two countries with the same language, affect the development of the reading. On the other hand, several studies have examined what skills are related to reading readiness (phonological awareness, alphabetic awareness, naming speed, linguistic skills, metalinguistic knowledge and basic cognitive processes), but there are no studies showing whether countries can also influence the development of these skills.Our main objective in this study was to establish whether there were differences in the degree of acquisition of these skills between Spanish (119 children) and Peruvian (128 children), five years old children assessed in their own countries and after controlling Economic, Social and Cultural Status (ESCS). The results show that there are significant differences in the degree of acquisition of these skills between these two samples. It's especially relevant, in these results, that the main predictor in a regression study was the country of origin, explaining a higher percentage of variance than other variables such as age differences, in months, or gender. These findings corroborate the results obtained in other studies with migrant population.

## Introduction

A large body of literature shows that literacy development models are quite similar, and they are influenced by social, cognitive, and linguistic variables that underlie differences in students’ pre-reading skills [1], [2].

### Early predictors of reading

Social, cognitive, and linguistic variables can contribute to explaining students’ differences in early literacy. In addition, these factors strengthen and interact with each other in a multiplicative effect [3].

Among the social variables, we have to highlight the role of socioeconomic status [4], [5], [6], [7], [8]. The most used index in education is the index of economic, social and cultural status (ESCS), based on the Programme for International Student Assessment (PISA). This indicator combines information on education and parents’ occupation with household possessions. The index is derived from three variables: the International Socio-Economic Index of Occupational Status (ISEI); the highest level of education of the student’s parents; the PISA index of family wealth; the PISA index of home educational resources; and the PISA index of possessions related to “classical” culture in the family home, include an item regarding the number of books a family has in the household. This has been shown in PISA studies as a predictor parameter of academic success [9].

It is commonly accepted that social and economic status play an important role in the acquisition and subsequent reading performance of students. In general, children from low-income families are more likely to have poor reading achievement [10]. The family income level, as well as the parent’s of education and occupation level, have a direct impact on the academic progress of their children [11], [12] just as unemployment or unstable occupation of parents can have a negative impact on the development of cognitive skills related to reading and literacy development [13], [14].

The family has been shown to influence the experiences that the home offers children, being these differences fundamental to the command of linguistic skills associated with emergent literacy. There really is an interaction between linguistic elements and shared reading experiences. The effect of the family on reading goes beyond these shared experiences, since the linguistic code used by the family is a determining factor in the acquisition of reading itself, the children's home plays a key role in mastering the language skills associated with reading initiation and development [15], [16], [17], [18]

On the other hand, the shared reading of a book contributes to the development of receptive language, which in turn is strongly linked to reading performance [18]. Likewise, a good pre-reading experience will be enriched by the child’s contact with elements related to written material [19]. Along these lines, studies have shown higher levels of reading competence in students whose parents have better attitudes toward reading and claim to spend more hours per week engaged on this activity [20].

In addition, some linguistic [21], [22] and metalinguistic [23], [24], [25] variables also facilitate emergent literacy.

Two of the linguistic skills are oral language components that are mainly related to reading acquisition: vocabulary, comprehensive and expressive [26], [27], and morphosyntactic development [28], [29]. These aspects of oral language are essential in reading acquisition, and they maintain their influence on reading comprehension during elementary education [22], [30].

Among the metalinguistic skills, phonological awareness can be highlighted as one of the most widely studied factor [31], [32], [23], [33], [34] as it is considered the main predictor of reading performance. Its acquisition helps to learn the alphabetic code, and this learning in turn significantly increases the acquisition of phonological awareness [35]. Its study is of great interest due to the reflexive posture of children towards oral language and the awareness of the units that conform written language [36]. Another relevant metalinguistic factor would be print knowledge: awareness of the reading objective [37], and the written word has meaning [38]. The child must be aware that writing is a representation which enables to perpetuate and transmit oral language [39]. The hypothesis that knowledge about written language is related to success in reading acquisition has received considerable support [40], [41], [42], [43], [44], [24], [45], [46].

It is relevant to point out that phonological, morphological, and orthographic awareness have significantly predicted reading success, even in non-alphabetic languages such as Chinese [47]. However, when comparing the predictors in transparent and opaque languages, studies have shown that in opaque languages, instruction time is necessary before phonological awareness and vocabulary begin to show their influence on reading acquisition [48].

In the case of alphabet knowledge [49], [50], [51], it facilitates literacy through the early acquisition of the correspondence between written letters and sounds [52].

Finally, within this group of predictors of reading performance, certain cognitive variables can be mentioned [53], such as attention [54], [55], perception [56], [57], and memory [58], [54], which act as mediators in learning to read [59].

### Evaluation of early predictors of reading in the Spanish language

Regarding the evaluation of these skills in Spanish-speaking children, various attempts have been made to measure them, both as individual and group assessment [60], [61], [62], [63], [64], [65], [66], [67]. Of the pre-reading tests published in Spanish, only three of them focus on evaluating these precursors: the Test for the early detection of reading and writing learning difficulties [60], the Pre-reading Skills Test [67], and the BIL (3–6), Batería de Inicio a la Lectura (Emergent Reading Battery) [66], for children from 3 to 6 years old. The latter includes the greatest number of skills and can be applied during the entire preschool period.

### Comparative studies on early predictors of reading

An important issue is whether the precursors of reading are the same in all languages and countries. Emergent literacy development models are quite similar, with identical cognitive and linguistic skills predicting reading [1], [2]. It is also necessary to ask whether the acquisition and development of these skills occur in a similar way in different countries.

Ethnic group seem to differentially predict the development of pre-reading skills in children [68]. In the study of Lopez-Escribano and Beltrán [68] examined emergent literacy skills in children with an average age of 5 years and 9 months (i.e., Knowledge about Print, Listening Comprehension, Receptive Vocabulary, Rapid Naming of Objects and Letters, and Phonological Awareness) in three groups of native Spanish speakers: Spaniards, Latin Americans, and Spanish Gypsies. As regression analyses indicated, ethnic background significantly predicted all the pre-literacy skills assessed, except listening comprehension. Mean scores showed that Spaniards outperformed the other two groups on the variables examined.

However, it is not known whether the influence of these differences between ethnic groups is due to comparing the migrant population to the local population. There are several researches that detect worse academic results in immigrant children than in native children [69], [70], [71], [72], [73] and in receptive vocabulary [74]. Similarly, on the PISA test, reading proficiency scores of students from immigrant backgrounds are consistently lower than those of native students [9], [75], with the reading performance of first and second generation immigrant students consistently lower than that of autochthonous students [76]. Therefore, the results found on that work could be due to the fact that a sample of local students was compared to a group of migrant students.

The main purpose of this study is to analyze whether the differences found by Lopez-Escribano and Beltrán [68] bweteen the Spanish (local students) and South American (migrant students), attributed to cultural differences, would be replicated by taking both samples in their country of origin

Lopez-Escribano & Beltran, [68] indicated that cultural background predicted significantly the pre-literacy skills assessed. Mean scores showed that Spanish students have better performance than the other two groups (South American migrants and gipsy people), although this study presented a serious problem, as Spanish students had been assessed in their country of origin as native population, while South American students belonged to a migrant population. Therefore, the differences between the two samples could have been due to the fact of comparing native and immigrant populations. The main hypothesis of our study is to try to prove that these differences remain when Spanish and South American students are all assessed in their country of origin, eliminating the possible influence of being part of a migrant group and maintaining controlled, as far as possible, socio-economic status of children in both countries.

The present study aims to analyze whether the results obtained in previous studies are due to cultural differences, or to belonging to the native population or the immigrant population. Therefore, to resolve this issue the participants are assessed in their own countries and that “Economic, Social and Cultural Status” is controlled. This allows to better explore if there are differences between Spanish and Peruvian children, and if these differences are due to other factors over an above the economic gap between Spain an Peru.

Specifically, this study has been developed between two countries from two different continents, Spain and Peru, but with the same language, although they have certain dialectal differences. Spanish, despite its large number of variants spoken in different countries, is considered a homogeneous language [77], [78]. This homogeneity is consolidated by institutional action, such as the Asociación de Academias de la Lengua (Association of Language Academies), and by the influence of the media [79]. This similarity between Spanish from Spain and South America is most pronounced in Peru. The language spoken in both countries is very similar, especially in the city of Lima, within the "Ribereña" area. It’s the Peruvian zone where the Spanish has remained more "pure", as it has received less influence from the indigenous languages. In this sense, it is worth mentioning that several authors attribute to Peruvian Spanish a great closeness to the tradition of Castilian Spanish. This idea was confirmed by Malmberg [80] when he asserted: "that Peruvian Spanish is, even today, the closest Spanish American language to the Castilian linguistic norm" (p. 140).

## Materials and methods

### Participants

The study was carried out with 119 Spanish (70 boys and 49 girls) and 128 Peruvian (67 boys and 61 girls) 5-year-old children. This study was approved by the Experimental Research Ethics Committee of the University of Valencia (Spain). We obtained written informed consent from their parents before participating in the experiment.

The Spanish sample comes from 8 schools located in different regions of Spain. The selection of these centers has taken into account the socioeconomic medium of their neighborhood. All Spanish schools belong to neighborhoods with a medium socio-economic level. The Peruvian sample comes from 4 centers of the city of Lima, these centers are located in especially "favoured" neighborhoods so they have a high socio-cultural level with respect to the Peruvian population, but quite similar to the Spanish schools. The Peruvian schools belonged to two Metropolitan Lima districts with similar socioeconomic characteristics and according to the wealth indicators analyzed [81], [82] both districts are located in quintile 5, which represents households with higher incomes compared to other areas of the country.

In addition, in the selection of the Peruvian sample, family data were obtained, based on the Questionnaire on the Socioeconomic and Cultural Status of the Family, indicators established in the Spanish PISA evaluations [9], [75]; taking into account the level of education and occupation of parents, as well as questions about possessions. The purpose was to obtain more detailed information from the ESCS in the Peruvian sample, to ensure that it was comparable to the Spanish population. These data show that in the Peruvian sample, parents have high levels of study, even higher than the Spanish averages, as can be seen in Table 1.

**Table 1:** Social and economic status.

**Parents’educational level**		
Country	Level	Percentage
	No studies	2%
Spain	Medium	55%
	High	45%
	No studies	1,1%
Peru (sample selected)	Medium	33,1%
	High	65%
**Parents’ Occupational Level**		
	Low	10%
Spain	Medium	47%
	High	43%
	Low	18%
Peru (sample selected)	Medium	56,3%
	High	25,7%

In both samples, the test was administered to all the children, with no selection or randomization process, in order to avoid any type of bias by the teachers or possible individual differences in pre-reading experience. Children were excluded if they had some type of specific educational need or their native language was not Spanish. In both countries the Early Childhood Education begins at the age of 3. This level is not obligatory in both countries but a high percentage of children are enrolled. Usually, around 100% of children at the age of 5 are schooled [83], [84].

### Variables and instruments

The variables used in the study were: country of origin (0 Spain, 1 Peru) and gender (0 female, 1 male) as dichotomous variables, and the age of the children codified in months.

The instrument used to evaluate the emergent literacy skills was: BIL (3–6), Batería de Inicio a la Lectura (Emergent Reading Battery) for children from 3 to 6 years old [36]. This instrument consists of fifteen tests grouped in five factors. In the study was been used the following tests:

The Phonological Awareness factor (PA) consists of 5 tests:

✓ Rimes (Rim). Identify whether the final sounds of pairs of words are the same or different (12 items).
✓ Word Count (WCo). The child has to indicate the number of words that make up each of the sentences presented. (6 items).
✓ Syllable Count (SCo). Requires dividing the word into syllabes. (14 items).
✓ Isolate Syllables (SIs). Recognize beginning phonemes or syllables of eight words (8 items)
✓ Omit Syllables (SOm). Leave out syllables in different positions. (5 items).

Alphabet Knowledge factor (AK). Consists of only one test that requires recognizing the name or sound of uppercase or lowercase letters (24 items).

Metalinguistic Awareness factor“Print Knowledge” (MA) is composed of three tasks:

✓ Word Recognition (WRe). A list of 10 written stimuli are presented, and the child must indicate whether or not each stimulus is a word (10 items).
✓ Sentence Recognition (SRe). Similar to the previous task, but a list of five stimuli are presented (5 items).
✓ Reading Functions (RFu). A visually represented sequence is narrated in four images in which the child must indicate how reading functions in the story (5 scenes).

Linguistic Skills (LS):

✓ Vocabulary (Voc). Name a set of pictures. (8 items).
✓ Basic Concepts (BCo). Recognition of concepts in drawings (8 items).
✓ Grammatical Structures (GSt). Recognize whether the syntax of a sentence is correct or not (6 sentences).

Cognitive Processes (CP) has two tasks:

✓ Auditory Sequential Memory (AsM). Repeat a set words in the same order (8 items). Maximum rank: 35, according to the number of words remembered and their position in the series.
✓ Visual Perception (VPe). Observe a model symbol and point to the ones that are the same (22 items).

The BIL is applied individually and takes about 25 minutes per child by native teachers in both countries. Because it is designed for preschool children, it relies on graphic stimuli. The items focus exclusively on the levels demonstrated prior to contact with reading, and low and medium difficulty tasks have been used in pre-reading children. The reliabilities indexes obtained, in the original report by authors, by the different tests that make up the BIL range from 0.54 to 0.97 (Table 2).

**Table 2:** Reliability index.

*Factors*	*Tests*	*Alfa Cronbach*
*Phonological Awareness*	Rimes	0,84
	Word Count	0,64
	Syllable Count	0,81
	Isolate Syllables	0,82
	Omisión Sílabas	0,73
*Alphabet Knowledge*	Alphabet Knowledge	0,97
*Metalinguistic Awareness “Print Knowledge”*	Word Recognition	0,77
	Sentence Recognition	0,69
	Reading Functions	0,72
*Linguistic Skills*	Vocabulary	0,69
	Basic Concepts	0,67
	Grammatical Structures	0,54
*Cognitive Processes*	Auditory Sequential Memory	0,88
	Visual Perception	0,87

Although the test uses a simple and basic vocabulary, it was decided to check, previously, the subtest of vocabulary, to ensure that the words used by the Peruvian children to name the elements of the test were the same as those used by Spanish children. In this group, it was found that when the child knew the word, he always used the same word as the one supposedly correct by the test. No child responded to any item with a synonym or specific word to their region. Based on these results, it was not considered necessary to revise the test or its correction criteria. Alongside these lexical varieties we reviewed all items of the test and found that two items of the alphabetical knowledge test and one item of the phonological awareness test could be affected by the phonological differences between the two dialectal variances. The most important differences are found in some phonological varieties of Spanish spoken in Peru: the /s/, /c/ and /z/ are pronounced the similar way; the /rr/ and /r/ are pronounced without fricativization; the "s" is predorsal and aspired before consonant (not at the end of syllable); the final /d/ becomes /t/ or is omitted; the sound /ll/ is pronounced as /y/; and tendency to eliminate hiatus in words with the suffix ‘–ear’.

In this case, we also tested these items empirically, using the same group of Peruvian children. We found two problems, in the letter “c” children said the name of this letter as ‘ce’ or ‘se’ (incorrect pronunciation) and the letter “z” was said as ‘zeta’ or ‘seta’ (incorrect pronunciation). In these two cases we decided consider any of these variations as correct. The Peruvian evaluators were urged to check the correct identification of the letter, not its pronunciation. In the third possibly problematic item, belonging to the phonological awareness test, there was no evidence of confusion with the letter /s/ at the beginning of a word, since when the evaluator pronounced the item correctly the children were able to isolate the sound without further difficulty. Based on this result no modification was considered necessary. Further, this original instrument to be supported by other Peruvian authors [85].

### Procedure

To analyze the data obtained, statistical tests were utilized that were similar to those used in previous studies with migrant and native populations in order to establish whether the significant differences between the two samples were due to being a migrant population. The study was conducted with a comparison of means, based on Student’s t test, in order to determine whether there were statistically significant differences between the children from the two countries. The Levene Test was used to test the homogeneity of the variances; if the differences in variances were significant, the t value was corrected. The effect size was also estimated for independent samples. The calculation is based on the formulas reported by Borenstein [86].

In addition, as in the study by López-Escribano and Beltrán [68], we performed a step-wise regression analysis to establish to what degree the country of origin, gender, and age predicted the degree of acquisition of the reading precursors analyzed, based on the main factors assessed by the BIL 3–6.

## Results and discussion

The results of the t tests performed indicate that there are significant differences between the two groups on the majority of the skills evaluated (Table 3).

**Table 3:** Results of t-test and descriptive statistics.

	**Country**						***Effect Sizes***	**Levene**	
	**Spain**		**Peru**						
	**Mean**	**SD**	**Mean**	**SD**	***t(df)***	***P***	***d***	***f***	***P***
*Phonological awareness tasks*	27.0	5.7	20.9	5.9	-8.158	.000	-1.061***	2.005	.158
					(235)				
Rimes	6.96	3.3	7.18	1.5	.669	.504	0.086	43.008	.000
					(158.546)				
Word Count	3.59	1.1	2.31	1.5	-7.640	.000	-0.977***	15.807	.000
					(234.180)				
Syllable Count	11.39	2.4	7.63	4.3	-8.521	.000	-1.085***	74.071	.000
					(204.360)				
Isolate Syllab & Phonemes	6.75	1.6	5.62	1.9	-5.099	.000	-0.657**	7.840	.006
					(238.897)				
Omit Syllables	3.80	1.1	1.94	1.5	-10.846	.000	-1.381***	27.426	.000
					(232.488)				
*Alphabet knowledge*	15.6	6.9	9.7	7.0	-6.652	.000	-0.851***	.069	.793
					(243)				
*Metalinguistic awareness tasks*	11.6	2.6	9.3	2.7	-6.657	.000	-0.851***	1.369	.243
					(243)				
Word recognition	8.44	1.9	7.12	2.2	-5.057	.000	-0.647**	3.278	.071
					(243)				
Sentence recognition	4.00	1.3	3.16	1.6	-4.636	.000	-0.593**	7.428	.007
					(239.918)				
Reading Functions	3.40	1.4	2.61	1.3	-4.679	.000	-0.596**	.216	.643
					(245)				
*Linguistic Skills tasks*	23.3	3.8	20.0	4.3	-6.258	.000	-0.800***	2.464	.118
					(237)				
Vocabulary	5.84	1.5	4.80	1.8	-4.957	.000	-0.635**	2.518	.114
					(243)				
Basic Concepts	6.55	1.4	5.7	1.3	-5.072	.000	-0.646**	.506	.478
					(242)				
Grammatical structures	4.52	1.3	4.06	1.3	-2.528	.012	-0.322*	.116	.733
					(245)				
*Cognitive processes tasks*	36.2	6.7	31.2	7.6	-5.405	.000	-0.690**	1.879	.172
					(244)				
Auditory sequential memory	26.60	6.2	26.02	5.3	-.781	.435	-0.099 -	3.814	.052
					(245)				
Visual perception	18.36	4.5	13.85	6.0	-6.748	.000	-0.861***	9.988	.002
					(234.069)				

Effect Sizes *** (Large Effect) ** (Medium Effect) * (Small Effect)–(No Effect)

Significant differences are detected on the majority of the skills studied, except on the “Rhyme” subtest of the “Phonological Awareness” test and the “Auditory Sequential Memory” subtest of the “Cognitive Processes” test. In addition, most of the tests present differences with a significance above 0.001, and large or medium effect sizes are found on all the significant variables, except Grammatical Structures, with a small effect size.

On the regression analyses performed using the BIL 3–6 factors as DV, the Country variable was in all cases the best predictor of the reading precursors studied, as its influence was even stronger than what was found between native and emigrant Latin American children living in Spain [68]. In the case of Phonological Awareness, the country of origin variable was highly significant, explaining 22% of the variance (S1 Table). These differences are more significant because this effect was not found in the emigrant population living in Spain. With regard to Alphabet Knowledge (S2 Table) and Metalinguistic Awareness (S3 Table), the country was significant, explaining 15% of the variance. In addition, Linguistic Skills (S4 Table), showed the greatest difference, with 38% of the variance explained by the Country of origin. These results are similar to those found by López-Escribano and Beltrán [68], where vocabulary explained 33% of the variance between Latin emigrant and Spanish children. Finally, on Cognitive Skills (S5 Table) the differences between countries were smaller, explaining only 10% of the variance. In all these analyses, in the next step of the statistical procedure, the age variable was introduced in second place, expect for the Metalinguistic factor, where Gender explained a larger percentage of the variance. The three variables used were always significant as predictors, explaining from 19.4% of the variance in the case of Cognitive Processes to 47.3% of the variance in the case of Linguistic Skills in the final model.

## Discussion

The main purpose of this study was to analyze whether the differences found by Lopez-Escribano and Beltrán [68] between the Spanish (local students) and South American (migrant students), attributed to cultural differences, would be replicated by taking both samples in their country of origin.

The data obtained shows significant differences between the emergent literacy skills of the two populations studied, corroborating the study by López-Escribano and Beltrán, [68], even, or especially, when the populations are found in their country of origin.

The results show the same previous trend, being replicated even with a slight increase in the variance explained by the country variable, although in this case the South American sample had not been taken from the migrant population, nor in disadvantaged neighborhoods. In this study both samples were taken in their country of origin, although the Peruvian sample was selected among the most favoured neighborhoods of Lima in order to achieve ESCS the most similar as that of the Spanish population.

The main reasons for these differences should be associated with non-linguistic variables, related to the development of reading precursors in different cultural contexts Within our study, the only explanatory data we have is the number of books in the family home. In the evaluation of the ESCS in the Peruvian sample, the indices obtained were appreciably lower than the mean values obtained in the Spanish population, despite the fact that it was a sample with a higher index of studies and high levels of wealth. Recent studies in Spain and Perú that have included the variable «number of books in the home» among the indicators of families’ socioeconomic status have demonstrated that access to a certain number of books favors phonological and metalinguistic awareness [87], [88], [89]. The exploration of books, alone or in the company of an adult promotes interest in reading [19] and the development of reading precursors [90], with higher rates of reading competence among students whose parents have better attitudes towards reading and claim to dedicate more hours per week to this activity [20].

Studies have shown that home experiences play a key role in the command of linguistic skills associated with emergent literacy. For example, the shared reading of a book contributes to the development of receptive language, which, in turn, is strongly linked to reading performance [18]. Likewise, a child will have a greater chance of gaining early literacy if s/he comes into contact with elements related to written materials [19]. Higher levels of reading performance have been confirmed in students whose parents have better attitudes toward reading and spend more hours on this activity, demonstrating the importance of the socio-familiar context of the student [20].

Therefore, another factor that could be explanatory is the “home literacy environment”, set of activities that can be done at home related to reading readiness, such as: read together with the child, letter and sound recognition, rhyming, developing appropriate vocabulary, modeling reading behavior. These activities, developed at home around reading, can be responsible for the cultural differences between both countries, in all pre-reading factors. For example, related to Alphabet Knowledge, the pre-reading factor that is more related to the familiar exposure to written texts has evidenced that children with a clear command of letter knowledge come from families that help them to learn the names of the letters by relating them to certain familiar objects [91].

These questions reveal the parents’ importance in the development of these skills by, not always intentionally, placing their children in contact with elements of written language. However, it is also important to consider the role of schools in these two contexts because all the children attended school. There are certain differences in the curricula of the two countries; in the Spanish schools, there is more specific intervention in these skills. Studies in populations of children have made clear that these actions can favor emergent reading [92], [93], [94], [95], [96], [35], even helping to reduce the percentage of subjects at risk of learning difficulties and increasing their academic achievement [97]. For example, instructing children in the manipulation of the language’s phonemes is effective because it facilitates reading acquisition, compared to instruction that pays no attention to the development of phonological awareness [98]. In the same direction, there are certain skills related to learning to read in which the Peruvian curriculum does not explicitly intervene, such as the approach to the written code, whereas in Spain it is one of the main reading objectives [99].

However, it has been observed that exposure to school practices alone is not sufficient to promote the development of literacy skills [100]. In this area, new studies will have to determine whether family and/or school patterns, and their possible interactions, are responsible for these differences.

Moreover, differences have been detected in the cognitive factors that are essentially "cultural free". However, the cognitive factors evaluated by BIL 3–6, especially the “Visual Perception” test, are strongly influenced by the child's exposure to written texts. The BIL 3–6 was developed under the assumption that cognitive factors are only an adequate predictor of reading when the stimuli used for its evaluation are elements close to written texts, so the “Visual Perception” test uses several alphabetic characters as stimuli, five of which belong to the Latin alphabet, used in the Spanish language. Obviously, children with greater alphabetical knowledge will be favored when performing this type of task. Therefore, in the BIL 3–6 this test cannot be really considered to be entirely free from cultural influence. The differences between countries found in the “Cognitive Processes” test are due to the visual task, no differences were found in the “Auditory Sequential Memory” test, based on oral language rather than written language.

In summary, the cultural context of the family and, specifically, children’s access to written material, can be one of the factors explaining differences in these skills. In addition, including these pre-literacy skills in the Early Childhood curriculum and, therefore, intervening at young ages, can be another factor. Although these differences have been corroborated among countries with similar linguistic contexts, it is necessary to carry out more exhaustive studies that show how the different social and cultural variables influence the development of these reading precursor, in order to boost the creation of appropriate contexts for learning this basic and important skill.

## Supporting information

S1 Table Summary of Hierarchical Regression Analysis for variables Predicting phonological awareness (N = 237). (DOCX)

S2 Table Summary of Hierarchical Regression Analysis for variables Predicting alphabet knowledge (N = 245). (DOCX)

S3 Table Summary of Hierarchical Regression Analysis for variables Predicting metalinguistic awareness (N = 245). (DOCX)

S4 Table Summary of Hierarchical Regression Analysis for variables Predicting linguistic skills (N = 239). (DOCX)

S5 Table Summary of Hierarchical Regression Analysis for variables Predicting cognitive abilities (N = 246). (DOCX)
