# Supplementary material for: The skills related to the early reading acquisition in Spain and Peru
Source: PLoS One. 2018 Mar 5;13(3):e0193450. doi: 10.1371/journal.pone.0193450 (PMC5837129; doi:10.1371/journal.pone.0193450)
Supplement: S1 Table — (DOCX) [file pone.0193450.s001.docx]

**S1 Table 1. Summary of Hierarchical Regression Analysis for Variables Predicting Phonological Awareness (N = 237).**

|  | **Model 1** | | | **Model 2** | | | **Model 3** | | |
| --- | --- | --- | --- | --- | --- | --- | --- | --- | --- |
| **Variable** | **B** | **SE B** | **β** | **B** | **SE B** | **β** | **B** | **SE B** | **β** |
| Country | -6.145 | 0.755 | -0.469*** | - 6.792 | .729 | -.519*** | -7.016 | .714 | -.536*** |
| Age (month) |  |  |  | 6.243 | 1.231 | .282*** | 6.163 | 1.202 | .279*** |
| Gender |  |  |  |  |  |  | -2.537 | .707 | -.193*** |
| *R^2^* | .220 | | | .297 | | | .334 | | |
| *F* change *R^2^* | 66.305*** | | | 25.703*** | | | 12.864*** | | |

Country is a dummy variable: Spain (0) serving as the reference group.

Gender is a dummy variable: female (0) serving as the reference group.

**p* < .05. ***p* < .01. ****p* < .001.
