# Supplementary material for: The skills related to the early reading acquisition in Spain and Peru
Source: PLoS One. 2018 Mar 5;13(3):e0193450. doi: 10.1371/journal.pone.0193450 (PMC5837129; doi:10.1371/journal.pone.0193450)
Supplement: S2 Table — (DOCX) [file pone.0193450.s002.docx]

**S2 Table 2. Summary of Hierarchical Regression Analysis for Variables Predicting Alphabet Knowledge (N = 245).**

|  | **Model 1** | | | **Model 2** | | | **Model 3** | | |
| --- | --- | --- | --- | --- | --- | --- | --- | --- | --- |
| **Variable** | **B** | **SE B** | **β** | **B** | **SE B** | **β** | **B** | **SE B** | **β** |
| Country | -8.878 | 0.884 | -0.392*** | - 6.645 | .875 | -.431*** | -6.609 | .865 | -.441*** |
| Age (month) |  |  |  | 5.542 | 1.483 | .218*** | 5.511 | 1.463 | .217*** |
| Gender |  |  |  |  |  |  | -2.370 | .856 | -.157** |
| *R^2^* | .154 | | | .200 | | | .225 | | |
| *F* change *R^2^* | 44.246*** | | | 13.960*** | | | 7.669** | | |

Country is a dummy variable: Spain (0) serving as the reference group.

Gender is a dummy variable: female (0) serving as the reference group.

**p* < .05. ***p* < .01. ****p* < .001.
