# Supplementary material for: The skills related to the early reading acquisition in Spain and Peru
Source: PLoS One. 2018 Mar 5;13(3):e0193450. doi: 10.1371/journal.pone.0193450 (PMC5837129; doi:10.1371/journal.pone.0193450)
Supplement: S3 Table — (DOCX) [file pone.0193450.s003.docx]

**S3 Table 3. *Summary of Hierarchical Regression Analysis for Variables Predicting Metalinguistic Awareness (N = 245).***

|  | **Model 1** | | | **Model 2** | | | **Model 3** | | |
| --- | --- | --- | --- | --- | --- | --- | --- | --- | --- |
| **Variable** | **B** | **SE B** | **β** | **B** | **SE B** | **β** | **B** | **SE B** | **β** |
| Country | -2.279 | 0.342 | -0.33*** | - 2.371 | .335 | -.409*** | -2.553 | .336 | -.440*** |
| Gender |  |  |  | -1.224 | .337 | -.210*** | -1.225 | .332 | -.210*** |
| Age (month) |  |  |  |  |  |  | 1.640 | .569 | .166** |
| *R^2^* | .154 | | | .198 | | | .225 | | |
| *F* change *R^2^* | 44.311*** | | | 13.194*** | | | 8.300** | | |

Country is a dummy variable: Spain (0) serving as the reference group.

Gender is a dummy variable: female (0) serving as the reference group.

**p* < .05. ***p* < .01. ****p* < .001.
