# Supplementary material for: The skills related to the early reading acquisition in Spain and Peru
Source: PLoS One. 2018 Mar 5;13(3):e0193450. doi: 10.1371/journal.pone.0193450 (PMC5837129; doi:10.1371/journal.pone.0193450)
Supplement: S4 Table — (DOCX) [file pone.0193450.s004.docx]

**S4 Table 4. Summary of Hierarchical Regression Analysis for Variables Predicting Linguistic Skills (N = 239).**

|  | **Model 1** | | | **Model 2** | | | **Model 3** | | |
| --- | --- | --- | --- | --- | --- | --- | --- | --- | --- |
| **Variable** | **B** | **SE B** | **β** | **B** | **SE B** | **β** | **B** | **SE B** | **β** |
| Country | -3.327 | 0.532 | -0.377*** | - 3.676 | .524 | -.416*** | -3.760 | .516 | -.426*** |
| Age (month) |  |  |  | 3.501 | .891 | .233*** | 3.540 | .876 | .236*** |
| Gender |  |  |  |  |  |  | -1.511 | .510 | -.171** |
| *R^2^* | .377 | | | .441 | | | .473 | | |
| *F* change *R^2^* | 39.162*** | | | 15.449*** | | | 8.786** | | |

Country is a dummy variable: Spain (0) serving as the reference group.

Gender is a dummy variable: female (0) serving as the reference group.

**p* < .05. ***p* < .01. ****p* < .001.
