# Supplementary material for: The skills related to the early reading acquisition in Spain and Peru
Source: PLoS One. 2018 Mar 5;13(3):e0193450. doi: 10.1371/journal.pone.0193450 (PMC5837129; doi:10.1371/journal.pone.0193450)
Supplement: S5 Table — (DOCX) [file pone.0193450.s005.docx]

**S5 Table 5. *Summary of Hierarchical Regression Analysis for Variables Predicting Cognitive Abilities (N = 246).***

|  | **Model 1** | | | **Model 2** | | | **Model 3** | | |
| --- | --- | --- | --- | --- | --- | --- | --- | --- | --- |
| **Variable** | **B** | **SE B** | **β** | **B** | **SE B** | **β** | **B** | **SE B** | **β** |
| Country | -4.497 | 0.921 | -0.327*** | - 5.598 | .910 | -.368*** | -5.572 | .894 | -.379*** |
| Age (month) |  |  |  | 5.974 | 1.549 | .231*** | 5.957 | 1.520 | .231*** |
| Gender |  |  |  |  |  |  | -2.872 | .885 | -.188** |
| *R^2^* | .107 | | | .158 | | | .194 | | |
| *F* change *R^2^* | 29.123*** | | | 14.868*** | | | 10.540** | | |

Country is a dummy variable: Spain (0) serving as the reference group.

Gender is a dummy variable: female (0) serving as the reference group.

**p* < .05. ***p* < .01. ****p* < .001.
